# Seasonal BMI Changes of Rural Women Living in Anatolia 

**DOI:** 10.3390/ijerph9041159

**Published:** 2012-04-02

**Authors:** Çiğdem Sabbağ

**Affiliations:** Food and Beverage Department, Adiyaman University, 02040 Altinsehir, Adiyaman, Turkey; Email: csabbag06@gmail.com; Tel./Fax: +90-416-223-3800

**Keywords:** seasonal obesity, rural women, Anatolia

## Abstract

Today, obesity is one of the most evident public health problems in many parts of the World and it is more common among women. Several factors are affecting women’s obesity, among these short term weight fluctuations, either gain or loss, cause severe health disorders, particularly in rural areas where seasonal activity differs significantly throughout the year. Since this case has not been studied in detail, our research focused on prevalence and probable causes of seasonal rural obesity among women in two rural areas of Turkey. The study was undertaken with 100 participants. One-way ANOVA and one-way repeated ANOVA tests were utilized for categorical, continuous and repeated variables as study contains groups with more than one and repeated variables. Overweight is more common in the 18–30 years and 50+ years groups, whereas the absence of obesity, except during winter of 2010 in the 50+ years of age group, is most probably due to the widespread occurrence of diabetes for this age group. The highest BMI values for all groups, which were 25.2 ± 3.39 for 2009 and 26.1 ± 3.40 for 2010, were determined in winter, because of minimum physical activity, while summer BMIs were 24.1 ± 3.39 in 2009 and 25.1 ± 3.35 in 2010. This decrease was most probably due to intense agricultural field work in both regions. The majority of the women claimed that their weight is balanced in summer but results revealed that participants did not lose all the weight which was gained during winter months although BMI showed a significant fall from spring to autumn.

## 1. Introduction

Changes and fluctuations in weight, particularly for obese subjects, are an indicative of health problems [1,2,3] and create severe health disorders if the changes happen in the short term [4]. The risks of obesity are well documented by several studies [4,5,6,7]. The prevalence of obesity, contrary to popular belief, is more common in low-income segments of the society due to consumption of low cost but energy-dense foods such as carbohydrates [8]. While Sobal [9] outlines the obesity prevalence among low income women, studies undertaken in Egypt and China manifested an opposite finding which was an increase of weight with increasing welfare, particularly for rural women, as they tended to give up working in the field with increasing household income [1,10,11]. In parallel to the above studies, intensity of overweight/obesity both in urban and rural areas exceeds malnutrition in 37 developing countries [12] e.g., more than 60% of the adults, being more common in women, are overweight/obese in Turkey [13]. The prevalence and effects of overweight/obesity in Turkey is in an increasing trend [14,15,16]. Overweight/obesity in urban areas is more studied than rural areas in Turkey [17,18]. Yumuk *et al*. [16] determined 70% of the adult population in Konya (Central Turkey) was overweight/obese, with a higher rate for women than men. This finding is supported with another study undertaken in a suburban area in Turkey which revealed more than one quarter of the women have obesity or overweight problems [19]. The causes of high overweight/obesity in the country and the World can be attributed to the better access to food, decreased physical activity, and the consumption of relatively cheaper but high energy bearing foods [18,20]. 

Studies on BMI (body mass index) are generally based long term trends. However in rural areas, seasonal changes in daily life due to intense field work in crop seasons significantly affect body weight. The repeated increasing and decreasing body weight in short terms (monthly or seasonally) unfortunately has a tendency for higher BMI. The reasons for short term fluctuation intensity should be taken into account for the prevention of obesity-related health problems. Thus, our study focused on the prevalence and causes of seasonal overweight and obesity in two rural areas of Turkey which are also representative of high overweight/obese zones of the country. The outcomes of the study can also be used for mitigating and/or protecting rural women from high BMI [21]. 

## 2. Methods

The seasonal BMI variation of women in rural areas of Central (Karapınar) and Southeastern Anatolia (Adıyaman) in 2009 and 2010 were studied. The study was undertaken at two villages in each town, namely Kuyucak and Doluca in Adıyaman (Southeastern Anatolia), and Yeşilyurt and Hasanoba in Karapınar (Central Anatolia). Obesity is a common phenomenon in both study sites [22]. A series of questions were asked by interviewers to each participant to record their eating habits and daily life along with demographic properties. 

The populations of the sites ranged from 2,300 (Yeşilyurt, Karapınar) to 890 (Doluca, Adıyaman). A total of 100 subjects, 25 at each site, were selected among women whom are working in farmers aged from 18–64 years, which is a common working age range for the regions [23]. Women were classified into four age ranges (18–30, 31–40, 41–50, 50+ years) for determination of age and BMI relation in agricultural activity because women participation to field activities decreases with age. Body weight and height for BMI measurement were taken in autumn, winter, spring, and summer of 2009 and 2010 for evaluating seasonal changes of subjects’ [24]. A calibrated precision balance and a stadiometer were used for weight, and height measurement which were undertaken with removed shoes and light clothing. BMI ranges are classified as follows: underweight for <18.5 kg/m^2^, normal for 18.5–24.9 kg/m^2^, overweight for 25–29.9 kg/m^2^, obese for ≥30 kg/m^2^ [24]. BMI evaluation analyses were performed with SPSS 17.0 statistical software.

The outdoor agricultural activities begin generally 15 days earlier in SE Anatolia (late February) due to earlier spring conditions. The agricultural activities are grazing, field preparation by hoeing, fertilizing, irrigation and harvesting. Due to second crop cultivation at both sides agricultural activities continue until late-autumn (harvest). However, SE. Anatolian women generally work 10–15 days longer because of more appropriate mild climatic conditions for grazing and crop cultivation than in C. Anatolia. Cereals and sugar beet are major crops in C. Anatolia (Karapınar) whereas cotton and cereals are dominant in SE. Anatolia (Adıyaman). Women said that they spent approximately 11 hours in the field for agricultural activities (Table 1). In addition to field activities, vegetables and fruits are also grown in house gardens between spring and autumn. Following intense field work in from spring to late autumn, women switch to a more sedentary life in winter. 

**Table 1:** Agricultural calendar and crops in project side.

Location	Start of Cropping Season	End of Harvest	Type of Agriculture	Major Crops	Animal Husbandry	Daily Working Hours
C. Anatolia	Early March	November, second crop	Irrigated	Sugar beet, wheat, maize	Small ruminants, grazing between Late March–Late November	10 hours in Spring, 12 hours in Summer and Autumn
SE Anatolia	Second half of February	Late October (orchards, cotton)	Rain fed, vegetables irrigated	Wheat, cotton, maize	Small ruminants grazing between Late February–Early December	

**Table 2:** Age, height, body weight of participants (means ± SD).

		C. Anatolia	SE. Anatolia
		Mean ± SD	Mean ± SD
Age (year)		43.9 ± 12.0	39.0 ± 12.9
Height (cm)		166.7 ± 7.2	168.7 ± 7.9
Literacy		23.7 (%)	12.5 (%)
Weight (kg)	Autumn 2009	68.6 ± 1.1	64.8 ± 9.4
	Winter 2009	72.8 ± 1.1	68.9 ± 9.4
	Spring 2009	71.3 ± 1.1	67.5 ± 9.3
	Summer 2009	69.8 ± 1.1	65.9 ± 9.6
	Autumn 2010	71.3 ± 1.1	67.2 ± 9.9
	Winter 2010	75.2 ± 1.1	71.3 ± 9.7
	Spring 2010	74.0 ± 1.1	70.0 ± 9.7
	Summer 2010	72.6 ± 1.1	68.9 ± 9.5

Following the recent introduction of irrigation in Karapınar (C. Anatolia), the annual income of households has significantly increased compared to the standard rainfed agriculture practices of Adıyaman. The annual income of women working in agriculture in C. Anatolia is roughly 5,000–6,000 USD whereas participants living in southeastern part earn 3,000–4,000 USD. However, the incomes of both sites are relatively lower than country’s mean annual gross domestic product of 10,000 USD [25]. Moreover, the rate of literacy, which also effects on nutrition habits, is still quite low for both sites, Southeastern Anatolia being less than Central Anatolia (Table 2). 

The weekly major food consumption (%) values are recorded at each site for each season for determination of eating trends (Table 3). Also, common calorie levels are provided for evaluating overall calorie intake of subjects (Table 3). 

**Table 3:** The major foods consumed (%) in study area and their average calorie values.

	Central Anatolia				Southeastern Anatolia				Average Calories/100 g
	Autumn	Winter	Spring	Summer	Autumn	Winter	Spring	Summer	
Wheat products	18	18	19	18	20	21	18	18	150–460
Corn	4	3	4	3	2	3	3	4	350–450
Rice	6	5	5	4	4	4	3	2	300–400
Pasta	14	15	16	13	9	9	8	8	360–413
Legumes	5	4	4	5	7	6	6	6	320–450
Poultry	4	5	6	4	5	5	5	5	250–360
Meat	7	8	8	7	5	5	6	5	350–520
Egg	2	4	4	3	3	4	4	4	80–270
Dairy products	9	8	8	9	8	6	6	7	100–150
Dried vegetables	2	3	1	1	2	3	3	2	300
Fresh vegetables	5	2	7	9	7	4	7	9	50
Potato	5	7	6	5	12	15	13	14	200
Dried Fruits	4	4	1	2	3	3	5	2	200
Oil	7	8	6	5	5	8	6	5	300
Fresh Fruits	4	2	2	9	5	2	4	7	65
Sweets	4	4	3	3	3	3	3	2	200
TOTAL	100	100	100	100	100	100	100	100	

One-way ANOVA were utilized for categorical and continuous variables as study contains more than one group with variable in order to avoid statistical errors. A one-way repeated measure of ANOVA is also employed defining the significance of seasons on BMI which is repeated variable of the study. 

## 3. Results

A total of 100 women, 25 women at each site, participated in the study. All participants are involved in agricultural practices. Subjects were grouped into four age ranges, namely 18–30, 31–40, 41–50 and 50+ years, as marriage and working in the field are based on the age ranges employed in the study. The average age of participants is 43.9 ± 12.0 years in C. Anatolia, and 39.0 ± 12.9 years in SE. Anatolia, with high illiteracy rates (Table 2). The low number of younger age women at both sites is most probably due to the younger generation’s decision to move to city centers for better job and education opportunities. 

The weekly diet at present is still dominated by high energy bearing carbohydrate foods since they can be obtained with low cost and/or produced by villagers (Table 3). Thus, more than 1/3 food consumption is centered on wheat products which are followed by dairy products in C. Anatolia. C. Anatolian women’s food consumption is also based on wheat products and potatoes, but with less dairy products (Table 3). The relatively high levels of livestock in C. Anatolia increased consumption of dairy products. Fresh vegetables are used as salad or an additive to main courses so their consumption is not as high as desirable for a healthy diet [24]. The use of oil at both sites was boosted in winter due to preparation of traditional oily stew foods such as dry beans, pilaf and potato [26]. Also, sugar is widely consumed in both regions for making deserts and used in tea. Daily tea consumption reaches 13–15 tea cups a day at both sites. However, except the increase in oil consumption in winter and availability of fresh vegetable and foods in summer, the overall food consumption patterns throughout year are somewhat same (Table 3). So, the average daily calorie intake throughout the year is quite similar in both sites (Table 2) which is approximately 3,000–3,500 calories/day. But, this figure is quite high for a sedentary life in winter since studies suggest 1,900–2,000 daily calorie intakes for the ones who had sedentary life [27]. 

**Figure 1:**
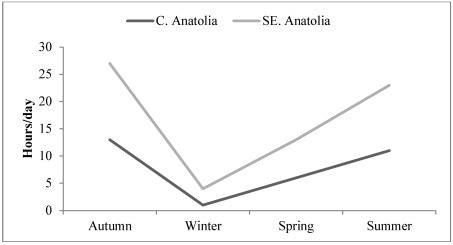
Daily hours spent in agricultural fields by C. and SE Anatolian women during the year.

Outdoor agricultural activities in C. Anatolia and SE Anatolia begin in early March/late February, respectively, and end in early November in the former and late October for the latter. Thus, women’s activity in the field, which as approximately 11 hours/day, reaches a maximum from the second half of summer to the second half of autumn (Figure 1). However, SE Anatolian women’s working hours are longer than those of CE Anatolian women due to the longer period of second crop cultivation. The major activity of women at the field for CE Anatolia is grazing and milking sheep, hoeing, irrigating and harvesting vegetables and fruits, whereas SE Anatolian women generally work in cotton and orchard fields for hoeing and harvesting. Also, most of the women grow vegetables and fruits in their house gardens for family consumption, which also increases daily working hours. 

**Figure 2:**
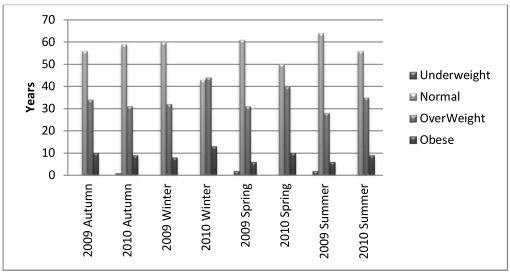
BMI changes of participants according to season and year.

Overweight is more common in the 18–30 years and 50+ years groups at both study sites (Figure 2). However, the number of overweight is less in the 18–30 years group than the 50+ years as teens are more responsible for housework and child care than elders throughout the year. With age, women’s activity in the field changes from physical activity to directing other women. 

**Figure 3:**
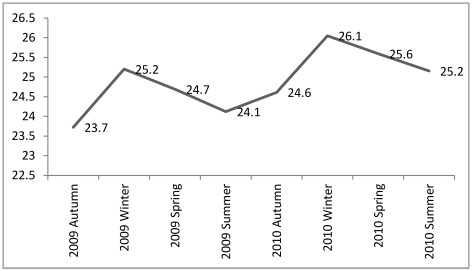
The mean BMI of participants in 2009 and 2010.

The main finding of the study is the significant seasonal body weight fluctuations of rural women in C. and SE. Anatolia. Women gain weight from autumn to early spring and lose weight from spring to autumn in both study sites (Table 2, Figure 3). The changes in BMI values with less than 1 may be evaluated as insignificant, however there is an increasing trend for the two years of study (Figure 2) it will negatively affect women health. The highest BMI is determined in winter with 25.2 ± 3.39 for 2009 and 26.1 ± 3.40 for 2010 when physical activity is at its minimum, and in summer BMI values were decreased to 24.1 ± 3.39 in 2009 and to 25.1 ± 3.35 in 2010 (Figure 2). The BMI exposed a significant fall from spring to autumn within the year, however the higher 2010 values than 2009 values (Figure 2 and Figure 3) manifested an insalubrious trend. The highest weight fluctuation throughout the year is observed at normal and overweight group members (Figure 2) notwithstanding the participants’ age.

**Figure 4:**
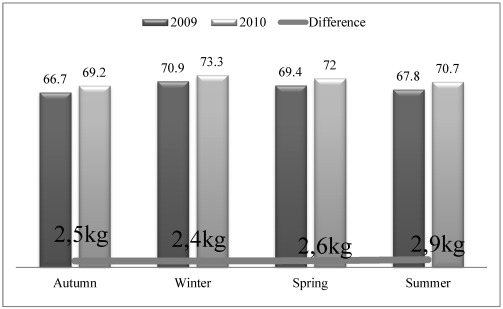
The mean body weight and annual differences of participants in 2009 and 2010.

Although women claimed that they lost the weight which they gained in winter due to the intensive outdoor agricultural activities from spring to fall, the measurements did not support this idea in light of an average permanent gain of 2.6 kg after the two years of the study (Figure 4). 

Studies revealed a positive relation with low socio-economic status and high BMI among women in rural areas [9]. However, in contrast to those, highest BMI and obesity prevalence were observed in Karapınar (C. Anatolia) than Adıyaman (S. Anatolia) where the former site has a relatively high income than the latter. We suggest that high income enables women to own more electronic appliances which in turn decrease their daily household duties as in the case in C. Anatolia [28]. 

**Table 4:** The BMI values of participants according to regions and seasons (*
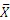
* : mean, sd: standard deviation).

		Autumn		Winter		Spring		Summer	
2009		* 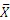 * ± sd	*p*	* 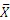 * ± sd	*p*	* 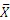 * ± sd	*p*	* 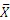 * ± sd	*p*
	Kuyucak	23.56 ± 3.34	0.107	24.93 ± 3.26	0.84	24.46 ± 3.20	0.109	23.76 ± 3.28	0.168
	Doluca	23.08 ± 3.07		24.63 ± 3.22		24.15 ± 3.23		23.61 ± 3.16	
	Yeşilyurt	23.08 ± 2.72		24.53 ± 2.66		24.03 ± 2.67		23.65 ± 2.76	
	Hasanoba	25.12 ± 4.09		26.68 ± 4.01		26.10 ± 4.07		25.43 ± 4.09	
2010	Kuyucak	24.24 ± 3.36	0.161	25.61 ± 3.44	0.209	25.21 ± 3.46	0.142	24.89 ± 3.42	0.247
	Doluca	24.06 ± 3.22		25.67 ± 3.13		25.13 ± 3.15		24.66 ± 3.03	
	Yeşilyurt	24.14 ± 2.80		25.58 ± 2.78		25.06 ± 2.63		24.71 ± 2.66	
	Hasanoba	25.97 ± 4.25		27.29 ± 3.99		26.95 ± 3.98		26.31 ± 4.04	

Moreover, the agricultural cropping is denser in SE Anatolia due to the five crops produced in two years which means more hours working in the field (Figure 1). Although the yearly BMI increase within each area is not statistically significant (*p* > 0.05), a positive relation exists with BMI increase and weight at each study site. The changes in BMI between years are significantly important at *p* < 0.01 level in C. Anatolia and SE Anatolia (Table 4). The repeated ANOVA analyses between each same months of 2009 and 2010 revealed a significant difference for the BMI (F: 376.698, *p* < 0.000) of participants which is in an increasing trend by years (Figure 3 and Figure 4, Table 4 and Table 5). 

**Table 5:** ANOVA results for 2009 and 2010 for BMI values among groups and between study sites.

	Source	Type III Sum of squares	df	Mean squares	F	*P*	Significant differences
2009	Between subjects	4,532.703	99	45.785	376.698	0.000	1-2, 1-3, 1-4, 2-3, 2-4, 3-4
	Measure	126.854	3	42.285			
	Error	33.339	297	0.112			
2010	Between subjects	4,524.508	99	45.702	158.324	0.000	1-2, 1-3, 1-4, 2-3, 2-4, 3-4
	Measure	113.587	3	37.862			
	Error	71.026	297	0.239			

## 4. Discussion

Agricultural practices in rural areas of Turkey are still labor intensive. Women participate in grazing, milking, weed control, pruning, hoeing, budding, cotton picking and harvesting (fruits and vegetables) activities which all require high physical power. Besides field activities, housework is also managed by women. Agricultural activities are intensified from spring to autumn in Turkey, whereas from late autumn to early spring, women shifted to a sedentary life in late autumn to early spring. The lower energy consumption during 5 to 6 months/year has a negative effect on body weight. Changes in physical activity are more effective on body weight than seasonal diet at two sites because diet patterns of both sites are centered on high carbohydrate containing and easily available wheat products, pasta and potatoes. The extreme seasonal physical activity changes in daily life, *i.e.*, from high energy consuming outdoor agricultural activities to sedentary life causes significant fluctuations in body weight in short term and mostly resulted an increasing BMI with years. Our study manifested the negative effect of seasonal fluctuations on BMI, a relatively short term in human life, due to intensity of physical activity and diet habits of women living in rural areas of C. and SE Anatolia of Turkey. Xavier and Sunyer [29] reported negative effects of fast weight loss and/or gain in short term with formation gallstone and cholecystitis, excessive loss of lean body mass, water and electrolyte issues, mild liver dysfunction, and elevated uric acid levels. The change of agricultural pattern from one crop/year to two to three crop/year due to the demand for high income generation increased field activities in both sides, and for meeting high physical activity energy requirements women tend to consume more carbohydrates and fatty foods, a trend which is also seen in other parts of the World [20]. The high carbohydrate containing food consumption in the study sites are attributed to two factors: traditional eating attitudes and the low socioeconomic status of rural women, both causing increasing prevalence of overweight/obesity [28]. 

Increasing BMI and obesity with age is a common problem in the World [1,3,30]. The increase of BMI in winter is common in rural areas where agricultural activities are at their minimum in the Northern Hemisphere [31]. Several studies have suggested obesity and high BMI in most parts of the World are linked to inequalities in education and income [3,5], and the tie between socioeconomic status and obesity reveals a more inverse relationship among women in developed societies [32]. Thus, the high illiteracy rate and low socio-economy along with traditional eating habits of women in rural areas of C. and SE Anatolian resulted in a high BMI. In contrast to previous studies’ findings which suggest a positive relation between low income and obesity, the relatively higher socio-economic status of C. Anatolian women has a positive correlation with high BMI and obesity, so the highest BMI and obesity prevalence were observed in Karapınar (C. Anatolia), whereas relatively low obesity and BMI were determined in Doluca (SE. Anatolia). This may be attributed to use of less electronic home appliances such as vacuum cleaners, washing machines and dishwashers by women in SE Anatolia than C. Anatolia [33]. 

The traditional diet at both sites is based on wheat products, lentils, chickpeas due to their local production for thousands of years. Traditional bread (free of salt and oil), lentils and chickpeas are healthy foods based on their mode of preparation. But, with the introduction of cash crops at both sites, the traditional cultivation of chickpeas and lentils is now negligible and their proportion in the daily diet is very low and eating patterns at both sites are now based on oily foodstuffs.

The most affected group for the prevalence of obesity and overweight is the 18–30 years group, which may be attributed to the heavy work load on the younger generations in the field which resulted in high fluctuations in weight gain and loss within a year. Moreover, some health problems such as diabetes [31] may be another effect on weight gains and losses in the 50+ years group but this assumption requires further detailed study in the research area. Thus, a health profession’s contribution is needed for further studies along with detailed information on daily type of food consumption

When participants are told for the danger of putting up weight in winter, they responded that they are losing weight in summer during the intense field activity. However, our results showed that majority of the women did not lose all the weight gained during winter months (Figure 3).

The high calorie containing food consumption of the 18–30 years age group in winter is most probably from their continuing diet habits which they used have in spring and summer when the physical activity is at its peak. The pattern of obesity and BMI in Karapınar and Adıyaman is in accordance with that seen in the women in Ghana where a higher time is devoted to agricultural tasks by women [34].

## 5. Conclusions

Women living in rural areas were until recently known to have low BMI than their counterparts in cities due to the high energy required for the intensive labor in the field. However, with the introduction of machinery for agricultural practice and home electronic appliances such as washing machines, vacuum cleaners for housework, women spend less working hours both at home and in the field. In addition to those factors, due to the lesser energy consumption in winter without changing the high calorie-containing diet from late autumn to early spring, the BMI of rural women in Turkey shows an increasing trend. Another risk is the severe effects of short term fluctuations of body weight *i.e.*, losses in cropping season, gains in winter season. Moreover, women believe that winter weight is easy to lose due to intense field work in spring and summer, but our study outcomes showed that winter weight is not totally lost during field activities as slight weight increases were determined after two years of study. The weight increase in general triggers development of several health disorders, particularly cardiovascular ones. However, the health problems need further study at both sites. Thus, women should be informed to take precautions for decreasing consumption of high carbohydrate containing foods in winter season which is a traditional diet. In the studied areas access to healthy foods such as vegetables is easy, however the difficulties in giving up traditional habits and gathering women for training courses in centers necessitates individual information activities like visiting women at their homes which can be organized via governmental health agencies or local administration bodies’ public service departments.
